# A systematic review and meta-analysis of the effects of different exercise modalities on frailty in older adults

**DOI:** 10.3389/fpubh.2026.1931299

**Published:** 2026-10-02

**Authors:** Jiahao Li, Huizhi Yang, Lili Chen

**Affiliations:** 1 Center for Rehabilitation Medicine, Rehabilitation & Sports Medicine Research Institute of Zhejiang Province, Department of Rehabilitation Medicine, Zhejiang Provincial People’s Hospital (Affiliated People’s Hospital), Hangzhou Medical College, Hangzhou, Zhejiang, China; 2 School of Sports Medicine and Rehabilitation, Beijing Sport University, Beijing, China

**Keywords:** frailty, older adults, resistance exercise, Tai Chi, walking

## Abstract

**Objective:**

To synthesize randomized evidence on unimodal resistance exercise, walking, and Tai Chi for frailty in older adults and to explore whether intervention characteristics may help explain between-study heterogeneity. Unlike broader reviews of multicomponent or non-pharmacological interventions, this review focuses on these three standalone modalities and reports modality- and dose-characteristic subgroup estimates.

**Methods:**

This systematic review and meta-analysis followed PRISMA 2020 and was prospectively registered in PROSPERO (CRD420251179862). Five databases were searched from inception to October 2025. Two reviewers independently screened records, extracted data, and assessed risk of bias with the Cochrane tool. Standardized mean differences (SMDs) were pooled using inverse-variance models; random-effects models were used when substantial heterogeneity was present. Prespecified subgroup analyses examined exercise modality and intervention characteristics. Leave-one-comparison-out sensitivity analysis was used to assess influence. Funnel-plot findings were considered exploratory because few comparisons were available.

**Results:**

Ten articles contributed 13 eligible exercise-control comparisons comprising 1,361 participant observations (682 intervention and 679 control). The overall random-effects estimate favored exercise (SMD = −0.57, 95% CI −0.91 to −0.24), but heterogeneity was substantial (*I*^2^ = 88%) and the approximate 95% prediction interval crossed the null (−1.73 to 0.57). Resistance-exercise comparisons showed a significant pooled association with lower frailty scores (SMD = −0.78, 95% CI −1.22 to −0.35), whereas walking and Tai Chi estimates were imprecise and not statistically significant. Leave-one-comparison-out estimates remained in the same direction (SMD range, −0.65 to −0.46), although heterogeneity remained high. Dose-characteristic subgroup findings were based on small numbers of comparisons and should be regarded as exploratory rather than as evidence of an optimal prescription.

**Conclusion:**

Resistance-exercise trials showed a significant pooled reduction in frailty scores, but the available evidence does not establish superiority over walking or Tai Chi because no direct head-to-head or network comparison was performed. High heterogeneity, imprecise modality subgroups, and sparse dose strata limit certainty. The dose-related findings should be used to generate hypotheses for adequately powered comparative trials rather than to prescribe an optimal regimen.

## 1 Introduction

With the rapid progression of global population ageing, frailty has emerged as a major geriatric syndrome threatening the health and independence of older adults (1). Frailty markedly impairs activities of daily living (2) and is strongly associated with sarcopenia, functional decline, and increased mortality (3). Epidemiological data indicate that the prevalence of frailty rises steadily with age, imposing a substantial burden on healthcare systems worldwide (1).

Exercise intervention is widely recognized as an important non-pharmacological approach for the prevention and management of frailty (4, 5). Physical exercise may address several frailty-related mechanisms by improving muscle strength (6, 7), cardiopulmonary function (4, 8), balance, and systemic signaling through exercise-induced myokines (5). Resistance training can increase muscular strength and physical performance (9). Aerobic exercise may improve functional capacity through cardiovascular and anti-inflammatory pathways (10). Tai Chi combines balance, flexibility, coordination, and mental regulation and may therefore affect frailty through neuromuscular and psychosocial pathways (11).

Earlier reviews have examined exercise broadly, multicomponent programs, or multiple non-pharmacological interventions, including network meta-analysis (1, 2, 7, 12). The present review addresses a narrower question by restricting synthesis to randomized trials of standalone resistance exercise, walking, or Tai Chi and by exploring intervention intensity, session duration, weekly frequency, and program duration. Its contribution is therefore a focused update and an exploratory characterization of exercise-delivery features, not a definitive ranking of modalities or doses.

## 2 Methods

This systematic review and meta-analysis was conducted and reported in accordance with PRISMA 2020. The protocol was prospectively registered in PROSPERO in October 2025 (CRD420251179862).

### 2.1 Search strategy and study selection

PubMed, the Cochrane Library, Embase, Web of Science, and EBSCOhost databases were searched from inception to October 2025. Search concepts covered older adults, resistance training, walking, Tai Chi, and frailty; controlled vocabulary and free-text terms were adapted for each database. The randomized controlled trial filter was applied where available. The complete PubMed strategy is reported in Table A1. Records were imported into EndNote and duplicates removed. Two authors (LJH and YHZ) independently screened titles/abstracts and full texts. Disagreements were resolved by discussion and, when necessary, consultation with a third reviewer.

### 2.2 Eligibility criteria

#### 2.2.1 Types of participants

Studies were eligible when participants were older adults aged at least 60 years and the study mean age was at least 65 years. Participants of any sex or nationality were eligible, provided they were not competitive athletes.

#### 2.2.2 Types of interventions

Eligible interventions were standalone resistance training, walking, or Tai Chi and were not combined with other exercise modalities or lifestyle components.

#### 2.2.3 Types of comparisons

Studies were eligible if the control groups were non-exercising or usual care.

#### 2.2.4 Types of outcomes

Studies were eligible if they reported at least one measure of frailty.

#### 2.2.5 Types of studies

Studies were eligible if they were randomized controlled trials (RCTs) published in English.

Studies were excluded if they were (1) duplicate publications; (2) reviews; (3) letters to the editor; (4) conference abstracts; or (5) animal studies. Reports were also excluded when the full text or analyzable outcome data could not be obtained. Eligibility and planned synthesis groups are summarized using PICOS in Table 1.

**Table 1:** Table PICOS criteria for inclusion and exclusion of studies.

Parameter	Defined criteria for the current study
P (population)	Older adults (aged ≥ 60 years), non-athletic
I (intervention)	Resistance training; Walking; Tai chi
C (comparison)	Usual care or sham training
O (outcomes)	Frailty
S (study design)	Randomized controlled trials

### 2.3 Data extraction

Two researchers independently extracted all data and compiled the results. In case of disagreement, a third researcher intervened. If relevant data were not available in the article, we contacted the authors to obtain the original data. We extracted the following information: lead author, year of publication; participant demographics; health condition, age, sample size of people in the experimental and control groups; exercise characteristics: type, frequency, duration, and intensity; and reported outcomes (frailty).

### 2.4 Quality and risk of bias assessment

Two reviewers independently assessed risk of bias using the Cochrane Risk of Bias tool across random-sequence generation, allocation concealment, blinding of participants/personnel, blinding of outcome assessment, incomplete outcome data, selective reporting, and other bias. Disagreements were resolved by consensus. Because participant blinding is generally infeasible in exercise trials, this domain was interpreted separately from assessor blinding. Funnel plots were examined only as an exploratory assessment of small-study effects because the number of comparisons was limited.

### 2.5 Data synthesis and analysis

Review Manager version 5.4 was used for quantitative synthesis. Mean differences were planned when studies used the same frailty scale; standardized mean differences (SMDs) with 95% confidence intervals were used when scales differed. Effects were oriented such that negative values indicated lower frailty after exercise. Fixed-effect models were used when heterogeneity was not substantial (*I*^2^ ≤ 50%); otherwise, inverse-variance random-effects models were used. Heterogeneity was assessed using Cochran’s Q and *I*^2^ and explored through prespecified subgroup analyses by modality, intensity, session duration, weekly frequency, and intervention duration. Because several strata contained only two or three comparisons, subgroup estimates were treated as exploratory and were not interpreted as proof of between-group differences. Leave-one-comparison-out sensitivity analysis assessed influence. When an article contributed more than one modality or follow-up comparison, each reported comparison was retained as in the original extraction; the resulting within-study dependence was considered when interpreting precision and is acknowledged as a limitation.

## 3 Result

### 3.1 Literature search and trial selection

The study-selection process is shown in Figure 1. The search identified 842 records. Before screening, 269 duplicate records and 83 reviews or meta-analyses were removed (352 records in total), leaving 490 records for title/abstract screening; 314 were excluded. Full texts of 176 reports were assessed, of which 166 were excluded for the following mutually exclusive reasons: inconsistent research content (*n* = 74), full text unavailable (*n* = 5), study protocols or methodological papers (*n* = 9), inconsistent outcome measures (*n* = 47), and inconsistent intervention types (*n* = 31). The remaining 10 articles were included in the qualitative and quantitative synthesis and contributed 13 eligible exercise-control comparisons.

**Figure 1:**
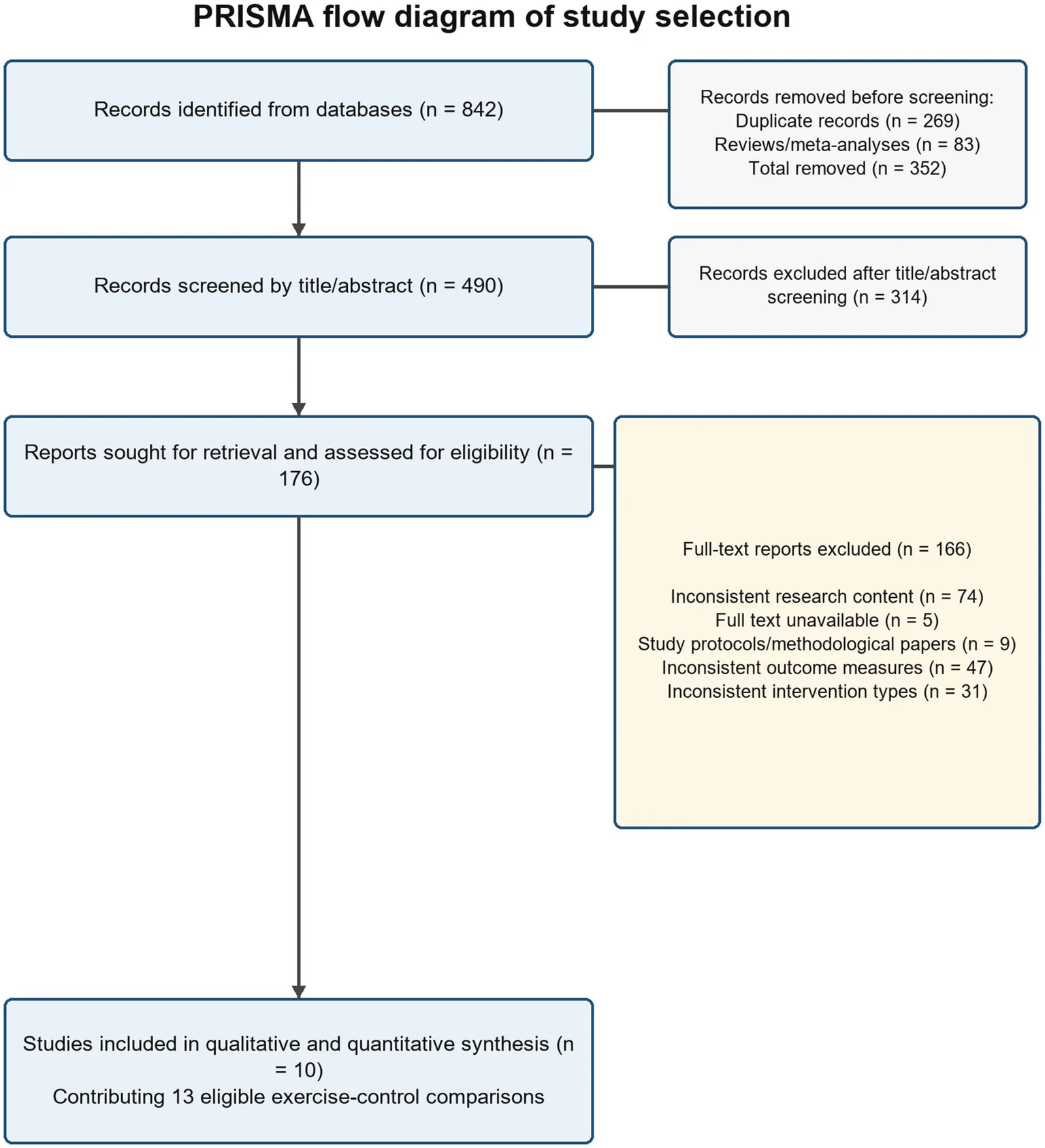
PRISMA flow diagram of study selection.

### 3.2 Description of the included trials

Ten articles contributed 13 exercise-control comparisons. Some articles reported more than one eligible modality or follow-up comparison; accordingly, the comparison count is larger than the article count. Across the extracted comparisons there were 1,361 participant observations (682 intervention and 679 control). Comparison sample sizes ranged from 12 to 185 analyzed participants, and mean participant ages ranged from approximately 70–101 years.

Exercise reporting varied across trials. Modalities comprised walking, resistance exercise, and Tai Chi; session duration ranged from 30 to 70 min when reported, weekly frequency from two to five sessions, and program duration from 4 to 48 weeks. Intensity was reported using percentage of one-repetition maximum, perceived exertion, heart-rate reserve, elastic-band color, or was not reported. These differences in intervention definition and reporting were considered potential sources of heterogeneity. Study characteristics and frailty instruments are shown in Table 2.

**Table 2:** Details of included studies.

Study	Participants	Sample size	Gender	Age	Exercise type	Intervention duration	Exercise frequency	Exercise intensity	Exercise duration	Outcome measures
Arrieta et al. (22)	Older adults in nursing homes	112	Male/Female	84.9 (70–102)	Resistance training	6 months	2 times/week	40 ~ 70% 1-RM	60 min	Fried
Barrachina-Igual et al. (23)	Older adults with frailty or pre-frailty	96	Male/Female	77.56 ± 7.51	Resistance training	20 weeks	2 times/week	After the initial adaptation phase (20–30% 1RM), progress to the target intensity (70–80% 1RM)	70 min	Fried
Kohn et al. (24)	Older adults with hypertension	167	Male/Female	72.1 ± 7.5	Tai Chi	12 weeks	2 times/week	N/A	60 min	Frailty index (FI)
Rujie Chen 2020 (1) (25)	Prefrail older adults	70	Male/Female	76.97 ± 5.19	Resistance training	4 weeks	3 times/week	Yellow elastic band	45–60 min	Fried
Chen et al. (25)	Prefrail older adults	70	Male/Female	76.97 ± 5.19	Resistance training	8 weeks	3 times/week	Yellow elastic band	45–60 min	Fried
Wu et al. (26)	Mild cognitive impairment (MCI)	68	Male/Female	70.12 ± 0.96	Resistance training	12 weeks	3 times/week	RPE10-14	30–50 min	Fried
Tou et al. (27)	Frail older adults	57	Male/Female	72.07 ± 8.14	Resistance training	12 weeks	2 times/week	N/A	60 min	Fried
Su et al. (28)	Healthy older adults	152	Male/Female	74.5 ± 7.1	Outdoor walking	12 weeks	2 times/week	N/A	60 min	Fried
Su et al. (28)	Healthy older adults	136	Male/Female	74.5 ± 7.1	Outdoor walking	21 weeks	2 times/week	N/A	60 min	Fried
Marcos-Perez et al. (13)	Long-lived older adults	19	Male/Female	101.4 ± 2.02	Resistance training	21 weeks	2 times/week	50 ~ 70% 1-RM	N/A	Fried
Liu et al. (29)	Frail older adults	146	Male/Female	80.75 ± 2.99	Tai Chi	12 months	5 times/week	N/A	40 min	Fried
Huang et al. (30)	Older adults with subjective cognitive decline (SCD)	209	Male/Female	72.3 ± 4.6	Walking	26 weeks	2 times/week	Weeks 1–2: 40%HRR; Weeks 3–8: 50% HRR; Weeks 9–12: 60% HRR; Weeks 13–26: 70% HRR	60 min	95-item frailty index (FI)
Huang et al. (30)	Older adults with subjective cognitive decline (SCD)	207	Male/Female	72.3 ± 4.6	Resistance training	26 weeks	2 times/week	Weeks 1–2: RPE 11; Weeks 3–12: RPE 13; Weeks 13–26: RPE 15	60 min	95-item frailty index (FI)

### 3.3 Methodological quality assessment

Risk-of-bias judgments are summarized in Figure 2. Randomization was reported across the included articles, although allocation concealment was not always clearly described. Blinding participants and personnel was generally infeasible and was judged high risk for the exercise comparisons. Only two articles clearly reported blinded outcome assessment; the remainder were judged unclear for this domain. Four articles had concerns related to attrition. No clear selective-reporting signal was identified, but absence of an available protocol for every trial limited certainty in this judgment.

**Figure 2:**
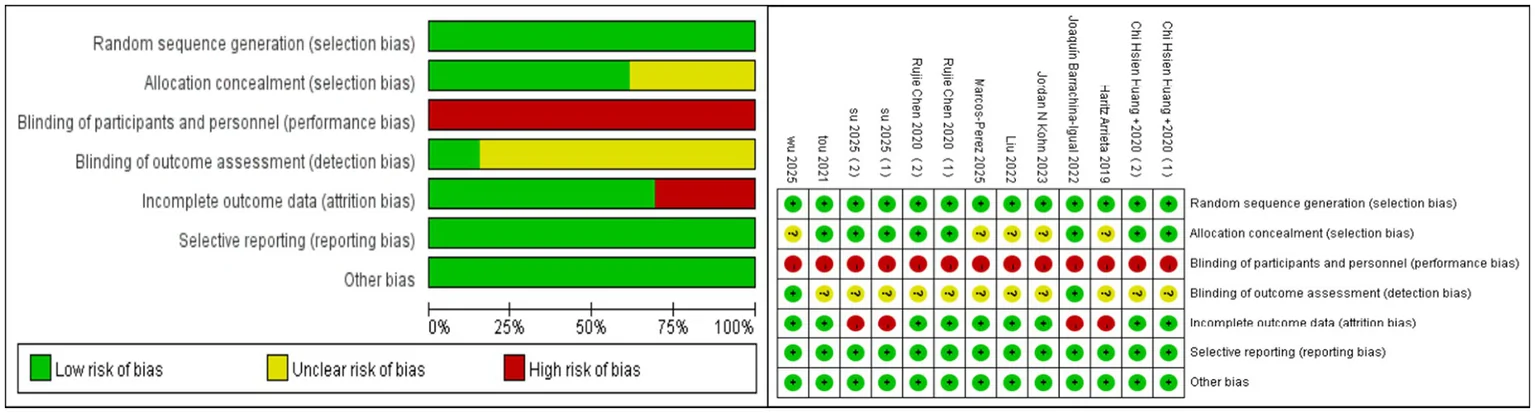
The risk assessment of bias by Cochrane.

### 3.4 Sensitivity analysis

Leave-one-comparison-out sensitivity analysis showed that the overall random-effects estimate remained in the same direction after each exclusion (pooled SMDs ranged from −0.65 to −0.46). However, I^2^ remained approximately 84–89%, indicating that no single comparison explained the substantial heterogeneity. Thus, the direction of the average effect was not driven by one comparison, but its magnitude and applicability remain uncertain.

### 3.5 Assessment of publication bias

The funnel plot appeared approximately symmetric (Figure 3). Because only 13 comparisons were available, this assessment had low power to detect small-study effects and should not be interpreted as evidence that publication or selective-reporting bias was absent.

**Figure 3:**
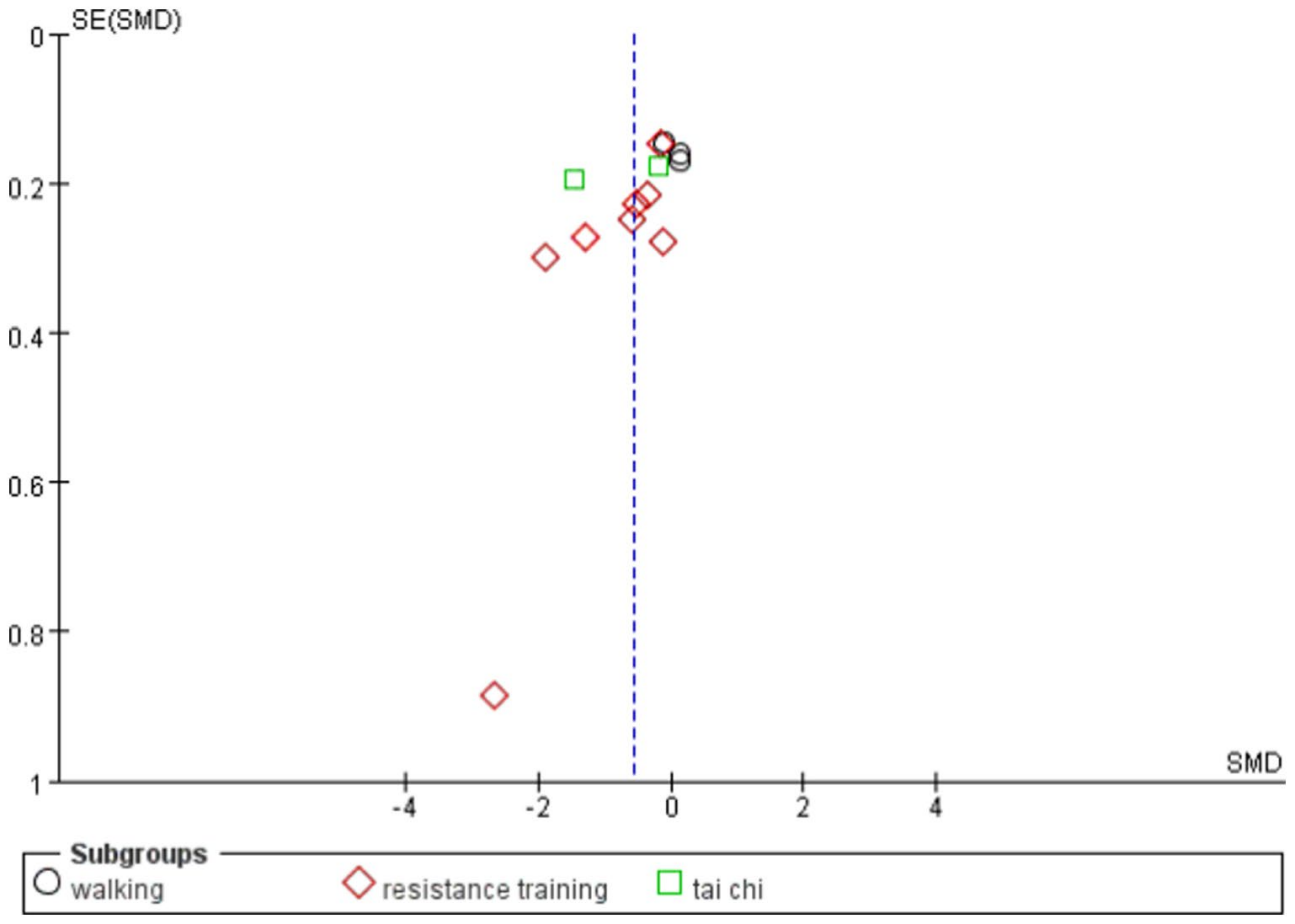
Funnel plots of publication bias.

### 3.6 Synthesis of the results

Figure 4 summarizes the prespecified subgroup analyses. Across all 13 comparisons, the random-effects estimate favored exercise (SMD = −0.57, 95% CI −0.91 to −0.24), with substantial heterogeneity (*I*^2^ = 88%); the approximate 95% prediction interval (−1.73 to 0.57) indicates that the effect in a new setting could plausibly include no benefit. By modality, walking included three comparisons (473 participant observations), resistance exercise eight comparisons (622 participant observations), and Tai Chi two comparisons (266 participant observations). Resistance-exercise comparisons had a significant pooled effect (SMD = −0.78, 95% CI −1.22 to −0.35), whereas walking and Tai Chi estimates were not statistically significant. This pattern does not demonstrate that resistance exercise is superior because modalities were not directly compared and the subgroup estimates differed in precision. Exploratory dose-characteristic analyses suggested significant pooled effects in the low- and moderate-intensity strata, sessions shorter than 60 min, frequency greater than 3 days/week, and programs shorter than 12 weeks; other strata were not statistically significant. Each dose stratum contained few comparisons, categories overlapped with modality and study characteristics, and no reliable dose–response or optimal prescription can be inferred from statistical significance within separate subgroups.

**Figure 4:**
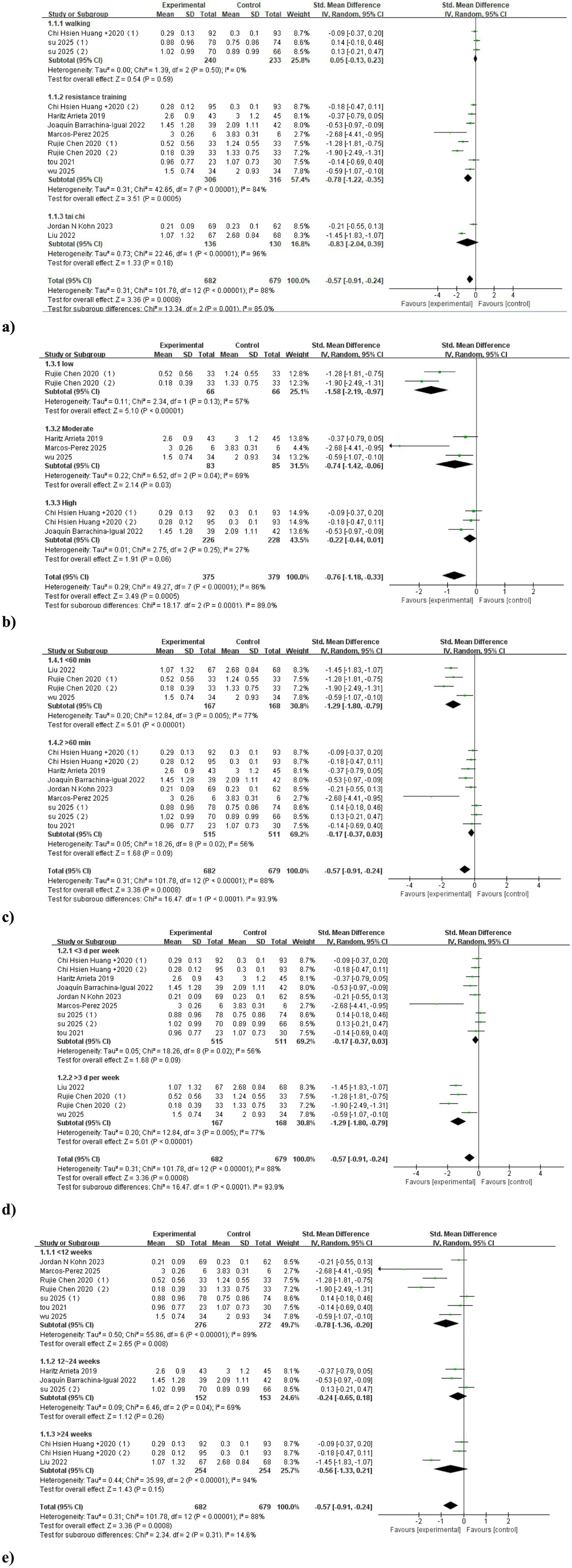
Forest plot of the meta-analysis. **(a)** Subgroup analysis by exercise type. **(b)** Subgroup analysis by exercise intensity. **(c)** Subgroup analysis by session duration. **(d)** Subgroup analysis by exercise frequency. **(e)** Subgroup analysis by intervention duration.

## 4 Discussion

This focused synthesis found that resistance-exercise comparisons had a statistically significant pooled association with lower frailty scores, while walking and Tai Chi estimates were not statistically significant. These are within-modality pooled estimates rather than direct comparative evidence; therefore, the findings should not be interpreted as a ranking of exercise modalities.

The findings are broadly compatible with prior reviews showing that exercise, particularly resistance-containing programs, can improve frailty-related function (1, 2, 7, 12). Resistance exercise plausibly targets weakness through mechanical loading, muscle protein synthesis, neuromuscular adaptation, and improved functional capacity (3, 4). Tai Chi and walking may provide balance, cardiovascular, psychological, and social benefits, but the present review contained only two Tai Chi and three walking comparisons, producing imprecise estimates. Observational evidence has also linked total and aerobic step counts with frailty outcomes in older adults with hypertension (31). Their non-significant pooled results should therefore not be interpreted as evidence of no benefit.

Potential mechanisms for resistance exercise include stimulation of muscle protein synthesis and satellite-cell activity, improved insulin sensitivity and mitochondrial function, modulation of inflammatory signaling, and enhanced neuromuscular coordination (3, 5, 13, 14). These mechanisms support biological plausibility but do not establish comparative superiority. Differences in baseline frailty, setting, supervision, adherence, intervention intensity, and outcome instruments may also modify observed effects.

Compared with broader systematic reviews and network meta-analyses (1, 2, 7, 12), this study offers a narrower update focused on standalone resistance exercise, walking, and Tai Chi and describes the exercise-delivery characteristics reported in randomized trials. Its practical contribution is to identify where evidence is relatively concentrated and where it remains sparse. Direct head-to-head trials or appropriately conducted network meta-analyses are required before modalities can be ranked.

The intensity subgroup results were exploratory. Low- and moderate-intensity strata had statistically significant pooled estimates, whereas the high-intensity stratum did not; however, each stratum included few comparisons, intensity definitions differed (percentage of 1-RM, RPE, HRR, or elastic-band color), and non-significance in one stratum is not evidence of a statistically significant difference between strata. The findings therefore generate a hypothesis that tolerable, progressive loading may be useful, consistent with prior dose–response reviews (15–17), but they do not define an optimal intensity.

The apparent pattern for shorter sessions and higher weekly frequency may reflect adherence, participant selection, modality, supervision, or other study-level factors rather than a causal dose effect. Research on older adults suggests that program burden can influence adherence (18, 19), but the present subgroup data cannot separate these mechanisms. The estimates should not be used as prescriptive thresholds for sessions shorter than 60 min or frequency greater than 3 days per week.

Similarly, the significant estimate in programs shorter than 12 weeks does not show that shorter programs are more effective than longer programs. Early neural adaptation may contribute to short-term improvement (20, 21), but longer trials may differ in population, intervention content, attrition, and outcome timing. Adequately powered trials with repeated follow-up and formal tests of interaction are needed to determine the durability and dose–response of exercise effects on frailty.

### 4.1 Heterogeneity and certainty of interpretation

Substantial heterogeneity persisted after leave-one-comparison-out analysis. Plausible contributors include modality, dose, setting, baseline frailty, age, supervision, follow-up time, and the use of Fried-based measures versus deficit-accumulation frailty indexes. Sparse and partly overlapping subgroups prevented reliable attribution of heterogeneity to a single factor. The wide prediction interval further indicates that the average pooled effect may not generalize to every clinical setting.

### 4.2 Strengths and limitations

Strengths include prospective registration, a five-database search, duplicate screening and extraction, restriction to randomized trials, and a focused comparison of standalone exercise modalities. Important limitations are the small number of comparisons within modality and dose strata; high residual heterogeneity; inconsistent definitions and reporting of exercise intensity; use of different frailty instruments (including Fried-derived scores and frailty indexes); inability to blind participants; attrition concerns; English-language restriction; low power to assess reporting bias; and possible within-study dependence when one article contributed multiple modalities or follow-up comparisons. Because subgroup differences were not established through adequately powered interaction tests or direct comparisons, all dose and modality interpretations should be considered exploratory. A formal GRADE Summary of Findings assessment was not performed, which further limits certainty statements.

## 5 Conclusion

Resistance-exercise comparisons showed a significant average reduction in frailty scores, but the evidence does not establish superiority over walking or Tai Chi. High heterogeneity and sparse, imprecise subgroups limit confidence in modality- and dose-specific estimates. Clinicians may consider progressive resistance exercise as one evidence-supported option while individualizing modality and dose to function, preference, safety, and adherence. Future trials should directly compare modalities, standardize frailty outcomes, report adherence and adverse events, and test dose–response with sufficient power.
